# Targeting Aging Skin with GABALAGEN^®^: A Synergistic Marine Nutricosmetic Ingredient Validated Through Human Randomized Trials

**DOI:** 10.3390/antiox14030245

**Published:** 2025-02-20

**Authors:** Jimin Hyun, Kyoung-Min Rheu, Bae-Jin Lee, Bomi Ryu

**Affiliations:** 1 Department of Food Science & Nutrition, Pukyong National University, Busan 48513, Republic of Korea; 2 Marine Bioprocess Co., Ltd., Busan 47281, Republic of Koreahansola82@hanmail.net (B.-J.L.)

**Keywords:** GABARAGEN^®^, GABA, collagen, fermentation, randomized trial, skin aging, nutricosmetics

## Abstract

This study introduces GABALAGEN^®^ (GBL), a marine-derived ingredient combining low-molecular-weight fish collagen and gamma-aminobutyric acid (GABA) produced via lactobacillus fermentation. GBL contains approximately 10% GABA, making up 39% of its free amino acid profile. A 12-week, randomized, double-blind, placebo-controlled trial with 100 adults (aged 35–60) assessed its effects on aging skin. Participants consumed 1500 mg/day of GBL in jelly form, with 94% completing the study. By Week 12, the GBL group showed a 20% increase in skin hydration and a 15% reduction in wrinkle depth. Improvements in skin density and elasticity were also observed, with no adverse effects reported. In vitro tests demonstrated strong antioxidant and anti-inflammatory effects, including enhanced superoxide dismutase activity and reduced pro-inflammatory cytokine expression in UVB-irradiated keratinocytes. GBL exemplifies sustainable innovation by upcycling fishery byproducts into high-value materials while addressing stability issues common to seafood-derived products. The fermentation process ensures safety and enhances GABA’s antioxidant activity and bioavailability. This scalable method aligns with circular economic principles and global sustainability goals, extending GBL’s potential to other functional materials which were proved their safety. GBL represents a breakthrough in nutricosmetics, combining efficacy, environmental sustainability, and industrial innovation.

## 1. Introduction

The overconsumption of resources and the mismanagement of waste have led to severe environmental pollution, posing one of the most critical threats to human health [1]. Among its many consequences, climate change and pollution have profoundly impacted skin health, the body’s largest organ and primary barrier against external aggressors [2]. Rising ultraviolet (UV) radiation, air pollution, and extreme temperature fluctuations accelerate skin aging and damage, manifesting as wrinkles, pigmentation, and reduced elasticity [3]. These environmental stressors not only compromise skin integrity but also contribute to a growing global burden of skin-related diseases, creating significant psychological distress for many individuals [4]. Recognizing this, the World Health Organization has identified climate change as a major global health threat [5].

Amid these challenges, sustainable consumption has emerged as a global priority, driving interest in eco-friendly solutions that mitigate environmental harm while delivering high-value benefits [6]. Marine bioresources have gained particular attention for their potential to address these dual objectives. By upcycling fish byproducts into functional materials such as low-molecular-weight collagen, these resources align with circular economy principles while offering superior bioavailability compared to land-based collagen sources [7]. Furthermore, combining marine collagen with bioactive compounds like gamma-aminobutyric acid (GABA), produced through microbial fermentation, presents an innovative approach to enhancing skin health [8].

Skin health research is particularly critical in this context due to the skin’s dual role as both a protective barrier and a mirror of internal health [9]. Chronic exposure to UV radiation and oxidative stress accelerates skin aging, which not only affects physical appearance but also impacts mental well-being [10,11]. In recent years, there has been a significant shift toward well-aging: a proactive approach to aging that emphasizes long-term health and vitality rather than merely addressing superficial signs of aging [12]. This shift has fueled the rapid growth of the nutricosmetics market, which bridges nutrition and cosmetics by offering ingestible products designed to enhance skin health from within. Projected to grow at an annual rate of over 8% by 2032, this market reflects increasing demand for scientifically validated, natural ingredients that align with sustainability and wellness trends [13].

In response to these trends, we tested the well-aging potential of GABALAGEN^®^ (GBL)—an innovative ingredient that combines low-molecular-weight fish collagen (FC) derived from fish scales with GABA produced through probiotic fermentation—at the pre-clinical level [8,14,15,16]. Furthermore, unlike previous studies that focused on single components such as collagen or GABA alone, this research investigates the synergistic effects of these two components on key indicators of skin aging such as hydration loss, reduced elasticity, and wrinkles. Through randomized trials, this study aims to establish GBL as a novel nutricosmetic solution that not only addresses consumer needs for well-aging but also aligns with global efforts toward sustainability by utilizing marine byproducts [17]. This research contributes to advancing both environmental responsibility and human wellness while setting a new standard in the nutricosmetics field.

## 2. Materials and Methods

### 2.1. Production of GBL

GBL was produced by Marine Bioprocess Co., Ltd. (Busan, Republic of Korea), a company affiliated with the authors who participated in the current research. Before fermentation, a fish-scale-byproduct-derived collagen from GELTECH Co., Ltd. (Busan, Republic of Korea) was enzymatically hydrolyzed at 55 ± 2 °C for 12 h with Prozyme 2000P (BISION-Biochem Inc., Gyeonggi-do, Republic of Korea). The FC production required two consecutive fermentations by *Lactobacillus brevis* BJ20 (accession No. KCTC 11377BP) and *Lactobacillus brevis* BJ21 (accession No. KCTC 18911P). A seed medium composed of these raw materials—1% yeast extract (Choheung, Ansan, Republic of Korea), 0.5% hydrated glucose (Choheung, Ansan, Republic of Korea), 1% L-glutamic acid (Samin chemical Co., Ltd., Siheung, Republic of Korea), and 98.5% water—was sterilized for 15 min at 121 ± 5 °C before being inoculated with 0.002% BJ20 and 0.002% *L*. *brevis* BJ21. These microorganisms were then cultured for 24 h at 37 °C separately. For the first fermentation, 10% (*v*/*v*) of the *L. brevis* BJ20 cultured seed medium was fermented in a fermentation medium (yeast extract 2%, glucose 0.28%, enzymatically hydrolyzed fish-scale-byproduct-derived collagen 29% (GELTECH Co., Ltd.), L-glutamic acid 5.5% (Samin chemical Co., Ltd.), water 63.22%) at 37 °C for 24 h. Then, 10% (*v*/*v*) of BJ21 cultured seed medium was added and fermented at 37 °C for another 24 h. The fermentation medium was sterilized and spray-dried to prepare GBL powder product (Lot. MBP-GABALAGEN-20230516). The ingredients including GBL, formulated in test and control supplements for randomized trials, are presented in the Supplementary Results (Table S2).

### 2.2. Free Amino Acid Analysis in GBL

The GBL was hydrolyzed and analyzed with an amino acid analyzer (L-8900, Hitachi Inc., Tokyo, Japan). Specifically, 10 mg of the GBL was hydrolyzed in 10 mL of 6.0 N HCl within a sealed vacuum ampoule at 110 °C for 24 h to determine its amino acid composition. After hydrolysis, the HCl was removed using a rotary evaporator, and the GBL sample was adjusted to a final volume of 10 mL. Amino acids were then quantified using the L-8900 amino acid analyzer (Hitachi Inc.). For free amino acid (FAA) analysis, 3.0 g of the GBL was mixed with an equal volume of 16% trichloroacetate solution and homogenized using a vortex mixer for 2 min. The homogenized sample was centrifuged at 3000 rpm for 15 min. Supernatants from two extractions were pooled and filtered through Whatman No. 41 filter paper (Whatman Inc., Florham Park, NJ, USA). The filtrate was acidified to pH 2.2 with a 10 M HCl solution and diluted to a final volume of 50 mL using distilled water. The resulting sample was analyzed using the same amino acid analyzer.

### 2.3. Cell Study

Human HaCaT keratinocytes were acquired from American Type Culture Collection (ATCC, Manassas, VA, USA). The cells were cultured in growth media consisting of Dulbecco’s Modified Eagle Medium (DMEM; Gibco, Thermo Fisher, Waltham, MA, USA), 10% fetal bovine serum (FBS, Gibco), and 1% penicillin–streptomycin (100 units/mL; Gibco) at a temperature of 37 °C with 5% CO_2_. To evaluate the cytotoxicity of GBL, HaCaT cells were cultured in 96-well plates at a density of 1 × 10^5^ cells/mL. After reaching full confluence, the cells were exposed to FCs at concentrations ranging from 1 to 100,000 μg/mL for 24 h. DMEM was replaced after treatment, and cells were gently washed with phosphate-buffered saline (PBS; Gibco). Subsequently, 10% of CCK-8 reagent (Dojindo Molecular Technologies Inc., Kumamoto, Japan) along with 0.1 mL of cultured medium was supplemented to each well. The plates were incubated at 37 °C for 1 h. The optical density of cell supernatants was measured at 450 nm using a microplate reader (Synergy HTX; Agilent Technologies Co., Ltd., Santa Clara, CA, USA). All of the data were investigated in triplicate to ensure consistent and reliable analysis.

#### 2.3.1. Enzyme-Linked Immunosorbent Assay

To evaluate the protective effect of GL against UVB irradiation, we determined the production levels of two representative biomarkers related to oxidative stress damage—8-hydroxy-2′-deoxyguanosine (8-OHdG) and superoxide dismutase (SOD)—using enzyme-linked immunosorbent assay (ELISA). HaCaT keratinocytes were grown to over 70% confluency. DMEM was then replaced with PBS (Gibco). Following that, the cells were exposed to 30 s UV (50 mJ/cm^2^) and treated with GBL (ranging from 50 to 350 μg/mL) for 48 h, and the cell cultured supernatants were collected for further analysis. Microplates were initially incubated overnight at 4 °C with 100 nM carbonate and bicarbonate mixed buffer (pH 9.6; Sigma-Aldrich, St. Louis, MO, USA) and then washed three times with PBS containing 0.1% Tween 20 (LPS solution, Daejeon, Republic of Korea) (TPBS) to eliminate unbound substances. To prevent nonspecific protein binding, the plates were blocked overnight at 4 °C with 5% skim milk (LPS solution) dissolved in 0.1% TPBS. After washing three more times with 0.1% TPBS, each well received 30 μg of cell supernatant sample and was incubated overnight at 4 °C. Following another wash with 0.1% TPBS, primary antibodies diluted in PBS (8-OHdG; sc-393871 and SOD; sc-515404, Santacruz Biotechnology Co., Ltd., Santa Cruz, CA, USA) were added to the wells and incubated overnight at 4 °C. After a final PBS wash, horseradish peroxidase-conjugated secondary antibodies (1:1000; Vector Laboratories, Burlingame, CA, USA) were introduced, and the plates were kept in the dark at room temperature for 4 h. Protein expression was visualized by adding tetramethylbenzidine solution (Sigma-Aldrich Co., Ltd., St. Louis, MA, USA) to each well and incubating at room temperature in the dark for 30 min. The reaction was terminated by adding sulfuric acid (Sigma-Aldrich), and the absorbance was measured at 450 nm with a microplate reader (Synergy HTX; Agilent Technologies Co., Ltd., Santa Clara, CA, USA). Absorbance values were normalized against a control group, and each experiment was conducted in triplicate for reliability and precision.

#### 2.3.2. Extraction of RNA and Quantitative Real-Time Polymerase Chain Reaction

RNA extraction and quantitative real-time polymerase chain reaction (qRT-PCR) were performed as described previously [18]. Homogenized muscle samples were treated with 500 µL of TRIzol (Takara Inc., Shiga, Japan), followed by the addition of chloroform (Sigma-Aldrich) to separate the clear supernatant (centrifuged at 12,000× *g*, 4 °C, for 15 min). RNA was precipitated by mixing the supernatant with 2-propanol (Sigma-Aldrich) and centrifuging at 12,000× *g*, 4 °C, for 10 min. The resulting RNA pellets were washed with 70% ethanol, air-dried at room temperature, and dissolved in diethyl pyrocarbonate (DEPC)-treated water (Sigma-Aldrich). The isolated RNA was reverse-transcribed into cDNA using M-MLV Reverse Transcriptase (Thermo Fisher). qRT-PCR was performed on a CFX 384 Touch™ Real-Time PCR Detection System (Bio-Rad, Irvine, CA, USA), and threshold cycle values were analyzed using CFX Manager™ software version 3.1 (Bio-Rad). Acbt served as internal control. The primer sequences for the target genes are provided in Table 1.

### 2.4. HPLC for the Indication Component Assessment in GBL

#### 2.4.1. Chemicals and Reagents

GABA and sodium acetate (50 mM, pH 6.5) were sourced from Sigma-Aldrich (USA). HPLC-grade acetonitrile, methanol, and distilled water (DW) were obtained from Samchun Pure Chemical Co., Ltd. (Pyeongtaek, Republic of Korea). Hydrochloric acid solution was purchased from Biosesang Inc. (Seongnam, Republic of Korea). Borate buffer (0.4 N in water, pH 10.2; Agilent P/N 5061-3339) and o-phthaldialdehyde reagent (10 mg/mL, Agilent P/N 5061-3335) were acquired from Agilent Technologies Inc. (Santa Clara, CA, USA). Acetic acid was supplied by Junsei Chemical Co., Ltd. (Tokyo, Japan).

#### 2.4.2. Standard Solution and Sample Preparation

To prepare a standard solution, 0.1 g of the standard sample was dissolved in 100 mL of DW using a volumetric flask. The resulting solution was filtered through a polytetrafluoroethylene syringe filter (25 mm/0.2 μm, Whatman Inc., Kent, UK) and stored at −80 °C. For the preparation of a 5% aqueous sample solution, 5 g of GBL was dissolved in DW in a 100 mL volumetric flask and similarly filtered through a polytetrafluoroethylene syringe filter (Whatman Inc., Kent, UK).

#### 2.4.3. HPLC Analysis Method

The analysis was conducted using a Dionex U3000 series HPLC system (Thermo Fisher Scientific) equipped with an ultraviolet (UV) detector operating at a flow rate of 1 mL/min. The samples were analyzed via UV–vis spectrophotometry at a wavelength of 338 nm. The GABA content in GBL was determined using the following formula:

$$Substance\left(mg/g\right)=\frac{measurement\left(mg/mL\right)\times dilution\;factor}{amount\left(g\right)}\times 100(mL)$$

### 2.5. Randomized Trials

#### 2.5.1. Study Design

This randomized trial (Approval No. M202301; 4 May 2023~23 November 2023) was a double-blind, placebo-controlled, single-center study designed to evaluate the effects of GBL on skin hydration and protection against UV-induced skin damage. The study adhered to Good Clinical Practice (GCP) guidelines and was approved by the Institutional Review Board (IRB). Participants were randomly assigned to either the experimental group (receiving GBL) or the control group (receiving a placebo) in a 1:1 ratio using block randomization following the randomization method. Specifically, the study employs a block randomization method to assign selected participants to the test and control groups in a 1:1 ratio. To maintain the integrity of the randomization process, a statistician arbitrarily or randomly determines the block size, which remains undisclosed to prevent prediction of group assignments. The randomization table is generated for approximately 120% of the target recruitment number. Participants are assigned unique three-digit identification codes (randomized numbers) in order of recruitment, according to the randomization schedule. The assignment of randomization numbers to subjects was performed without the involvement of guardians. They received either the test or control product packaged corresponding to their assigned code. Once a randomization number is assigned to a participant, it cannot be reused, even if that participant withdraws from the study. The statistician generates the randomization table and provides it to the sponsor. The sponsor then seals each participant’s randomization details in separate opaque envelopes and supplies them to the principal investigator for secure storage and management. This process ensures the double-blind nature of the study is maintained throughout its duration.

#### 2.5.2. Participants for Randomized Trial

The study recruited 100 healthy adults (men and women) aged 35–60 years with visible wrinkles in the crow’s feet area (Grade ≥ 3 based on expert visual assessment) and low skin hydration levels (Corneometer^®^ value ≤ 49). Participants were excluded if they had uncontrolled hypertension, diabetes, liver or kidney dysfunction, chronic diseases, or were using medications or supplements that could impact skin health within the specified washout period. Pregnant or lactating individuals were also excluded. More detailed exclusion criteria are presented in the Supplementary Material (Table S1).

#### 2.5.3. Intervention and Dermatologic Outcomes Measurement

Participants in the experimental group consumed GBL in stick jelly form (1500 mg/day) once daily for 12 weeks. The control group received an identical placebo jelly containing dextrin (1500 mg/day) (Table S2). Both products were indistinguishable in taste, appearance, and packaging to maintain blinding. The primary outcome was skin hydration, measured using Corneometer CM 825 (Courage + Khazaka Electronic GmbH Inc., Köln, Germany) at baseline, 6 weeks, and 12 weeks. The secondary outcomes were as follows: wrinkle severity, assessed via expert visual grading and 3D imaging using PRIMOSCR (Canfield Scientific Inc., Parsippany, NJ, USA); skin elasticity, measured with Cutometer MPA 580 (Courage + Khazaka Electronic GmbH Inc., Köln, Germany); transepidermal water loss (TEWL), evaluated using Vapometer (Delfin Technologies Co., Ltd., Kuopio, Finland); skin density, measured with DUB Skin Scanner (Courage + Khazaka Electronic GmbH Inc., Köln, Germany); skin roughness, quantified via PRIMOSCR imaging (Courage + Khazaka Electronic GmbH Inc., Köln, Germany); skin gloss, measured with Skin Gloss Meter (Delfin Technologies Co., Ltd., Kuopio, Finland); skin desquamation, analyzed using Visioscan VC 98 (Courage + Khazaka Electronic GmbH Inc., Köln, Germany).

##### Skin Wrinkles Assessment

Skin wrinkles were evaluated using a three-dimensional (3D) optical skin imaging device (Canfield Scientific Inc., Parsippany, NJ, USA). Parallel projection stripes were projected onto the skin in 3D; variations in stripe displacement, caused by differences in skin surface height, were quantified by the device’s software. Images captured before and after each measurement were matched for an identical reference area, enabling comparative analysis of skin wrinkles. Subjects were instructed to cleanse their face and rest under constant temperature (22–24 °C) and humidity (45–55%) conditions for 30 min prior to measurement. Additionally, they were asked to refrain from consuming water for 1 h before the test. The crow’s feet area was scanned, and the resulting images were analyzed to collect the following parameter values: Ra, Rmax, Rp, Rv, and Rz.

##### Measurement of Subsurface Skin Hydration

Subsurface skin hydration was assessed using a Moisturemeter D Compact (Delfin Technologies Co., Ltd., Kuopio, Finland). This instrument transmits a 265 MHz high-frequency electromagnetic field to a depth of approximately 2–2.5 mm beneath the skin surface and measures the reflected signal. The dielectric constant of the targeted tissue is converted into a percentage value of partial water content (PWC, %) for quantitative evaluation. Prior to the assessment, participants cleansed their faces, rested for 30 min under controlled temperature (22–24 °C) and humidity (45–55%) conditions, and refrained from fluid intake for at least 1 h. Three measurements were taken at the perpendicular intersection between the lateral canthus and the nasal bridge, and the mean of these three values was used for analysis.

##### Skin Elasticity Measurement

Skin elasticity was evaluated using a Cutometer MPA 580 (Courage + Khazaka Electronic GmbH Inc.). A negative pressure of 450 mbar was applied with an on/off time of 2.0 s, and measurements were taken three times. During each measurement, an infrared sensor determined the length of the skin drawn into a 2 mm diameter probe, allowing calculation of the elasticity parameters. Prior to measurement, subjects washed their faces and then rested for 30 min under controlled temperature (22–24 °C) and humidity (45–55%). In addition, water intake was restricted for 1 h before measurement. The measurement site was defined as the perpendicular intersection of the lateral canthus and the tip of the nose. Among the elasticity parameters (R0–R9) derived from the device, R2 (Ua/Uf), R5 (Ur/Ue), and R7 (Ur/Uf) values were used for analysis.

##### Measurement of TEWL

TEWL was measured using a Vapometer (Delfin Technologies Co., Ltd.). The device features a cylindrical measurement chamber equipped with sensitive humidity sensors, which monitor the increase in relative humidity (RH) within the chamber during the measurement process. The system automatically calculates the rate of water evaporation. Participants were instructed to wash their faces and then rest under controlled conditions of temperature (22–24 °C) and humidity (45–55%) for 30 min before the measurement. To minimize variability, participants were restricted from consuming water for at least 1 h prior to the assessment. TEWL was measured at the intersection of the lateral canthus and the tip of the nose. Each measurement was performed three times, and the average value was used for analysis.

##### Skin Density Measurement Method

Skin density was assessed using the DUB Skin Scanner (Courage + Khazaka Electronic GmbH Inc.), which operates with a 22 MHz ultrasound transducer. This device emits short electrical pulses to evaluate the density of the epidermis and dermis layers. Participants underwent the measurement under controlled environmental conditions of 22–24 °C temperature and 45–55% humidity. Prior to the assessment, participants washed their faces and acclimated to the controlled conditions for 30 min. To ensure accuracy, participants were instructed to refrain from water intake for at least 1 h before the measurement. The measurement focused on a point located 3 cm from the outer corner of the eye. Images captured during this process were analyzed to collect skin density values, providing a quantitative assessment of skin structure integrity.

##### Skin Roughness Measurement

Skin roughness was assessed using an optical 3D skin imaging device (Canfield Scientific Inc.). This device projects parallel projection stripes onto the skin surface, and the deformation of these stripes due to variations in skin height is quantitatively analyzed by a computer. Pre- and post-measurement images were matched to ensure consistent analysis of the same skin area. The measurement procedure was conducted under controlled temperature and humidity conditions (22–24 °C, 45–55%). Participants were instructed to refrain from water intake for at least 1 h prior to the measurement. After cleansing their faces, they acclimated to the controlled environment for 30 min before testing. Images of the cheek area were captured and analyzed, focusing on the Ra parameter value as the primary metric for skin roughness.

##### Skin Gloss Measurement

Skin gloss was measured using a Skin Gloss Meter (Delfin Technologies Co., Ltd.). This device is equipped with a 635 nm red semiconductor diode laser that projects a laser beam onto the skin surface and analyzes the light reflected at the same angle. Subjects washed their faces and then acclimated for 30 min in a temperature- and humidity-controlled environment (22–24 °C, 45–55% relative humidity) before measurements were taken. Water intake was restricted to 1 h prior to measurement. The measurement site was at the perpendicular intersection of the eye corner and the tip of the nose. Three measurements were taken at this site, and the average value was used for analysis.

##### Skin Desquamation Measurement

Skin desquamation was assessed using the Visioscan VC 98 (Courage + Khazaka Electronic GmbH Inc.). The measurement was performed by collecting skin scales using a special film (D-squame disk). The collected sample was then imaged and analyzed with the Visioscan VC 98. The Desquamation Index (D.I., %) obtained from this analysis was used as the evaluation parameter for skin desquamation.

#### 2.5.4. Data Recruitment Procedures for Study

Participants attended four visits: Screening Visit (1st visit, −21 to 0 days): informed consent, demographic data collection, physical examination, and baseline assessments; Baseline Visit (2nd visit, Start, Week 0): randomization, initial product distribution, and baseline measurements; Interim Visit (3rd visit, Week 6 ± 7 days): compliance check, adverse event monitoring, and interim assessments; Final Visit (4th visit, Terminal, Week 12 ± 7 days): final measurements of all outcomes and safety evaluations. The significance of skin health improvement following GBL intake was presented by comparing the start (2st visit) and terminal (4th visit) points.

#### 2.5.5. Interruption of Participants

Participants could be excluded from the study without their consent under the following circumstances: A. violation of inclusion/exclusion criteria; B. participant’s request to discontinue the trial; C. occurrence of adverse events (including serious adverse events); D. participant non-compliance (below 80%) or inability to observe; E. violation of the human trial protocol; F. consumption or need to consume medications or health functional foods that may affect the human trial; G. failure to take the test food for more than 5 consecutive days; H. other reasons based on the researcher’s judgment. Once a participant’s involvement in the study was terminated prematurely, they were supposed to be tested to ensure their safety. This precautionary measure was designed to protect the participant’s well-being after withdrawal from the study.

#### 2.5.6. Safety Assessments

Adverse events were monitored throughout the study via participant interviews and laboratory tests conducted at baseline and Week 12. Blood samples were analyzed for glucose, liver enzymes (SGOT/SGPT), creatinine, cholesterol levels, and other safety markers (Table S3).

#### 2.5.7. Statistical Analysis

The statistical analysis plan adopts the results of the Per Protocol (PP) analysis as the primary method for evaluating efficacy, while also conducting an analysis of the Full Analysis Set (FAS) group for reference. For the definition of analysis sets, subjects included in the efficacy evaluation for PP analysis must meet specific criteria. These include being part of the FAS group and completing the randomized trial up to Visit 4 without any significant protocol violations. Additionally, subjects included in the FAS analysis must have consumed test food at least once and have data available for primary efficacy variables after consumption. All statistical significance tests were conducted at a 5% significance level. The primary analysis of data obtained from study participants was performed on the PP set, with additional analyses conducted on the FAS set. For demographic data analysis, demographic variables measured at Visit 1 were summarized for each group and compared between groups. Continuous variables were tested using *t*-tests or Wilcoxon’s rank-sum tests, while categorical variables were analyzed using Chi-square tests or Fisher’s exact tests.

Efficacy analyses evaluated baseline characteristics and functional assessment data using both FAS and PP. The FAS includes participants who underwent at least one functional assessment after consuming the test or control food, while PP includes participants who adhered to the randomized trial protocol to a certain extent and completed the study. Safety evaluations will target the Safety Set (SS), which includes all participants who consumed the test or control food at least once after randomization. The normality of data was assessed using the Shapiro–Wilk test. For continuous variables, intergroup comparisons were analyzed using methods such as ANOVA, ANCOVA, or Kruskal–Wallis tests, with post hoc tests conducted if significant differences were found. Intragroup comparisons before and after intervention used paired *t*-tests or Wilcoxon’s signed rank tests. Outliers identified before unblinding might be considered in analyses. Adjustments for confounding factors, stratified analyses, and multiple comparison analyses were also performed as necessary. Categorical variables were compared between groups using Chi-square tests or Fisher’s exact tests. ‘*p*-value < 0.05’ was considered statistically significant. If there were differences in alcohol consumption history that could affect primary efficacy variables, covariate analyses controlling for this variable were additionally conducted.

## 3. Results

### 3.1. Production Process and Analytical Characterization of GBL

**Figure 1:**
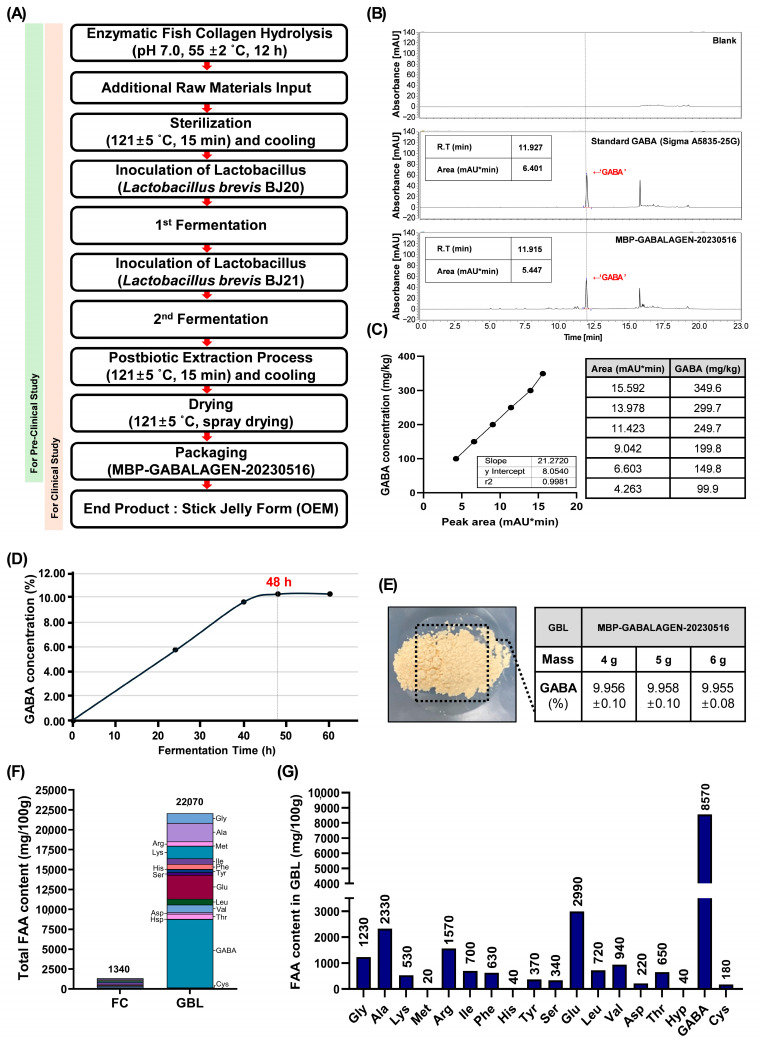
Development and validation of the GBL product. (**A**) The industrial mass-production process of GBL. The spray-dried GBL product, manufactured through a large-scale production process, was evaluated for its anti-skin-aging effects at the in vitro level. At the clinical level, GBL was provided in the form of stick jelly to examine its protective role against photo-induced skin aging. (**B**) The chromatograms of the marker component in GBL. GABA: Gamma-aminobutyric acid; R.T: Retention time. (**C**) Calibration curve of GABA standard material. (**D**) Time-dependent GABA production during fermentation process of GBL production. (**E**) The verified GABA content in GBL product. (**F**) Comparison of FFA content in FC and GBL. (**G**) The FFA profile in GBL. Gly: Glycine; Ala: Alanine; Lys: Lysine; Met: Methione; Arg: Arginine; Ile: Iso-leucine; Phe: Phenylalanine; His: Histidine; Tyr: Tyrosine; Ser: Serine; Glu: Glutamine; Leu: Leucine; Val: Valine; Asp: Aspartic acid; Thr: Threonine; Hyp: Hydroxyproline; Cys: Cystine.

Environmental pollution and climate change pose significant threats to human health, particularly affecting skin integrity. These stressors accelerate skin aging and contribute to various skin-related diseases. In response, sustainable consumption has become a global priority, with marine bioresources gaining attention for their eco-friendly potential. In our research, GBL, combining marine collagen derived from fish scale byproducts with bioactive compounds such as GABA produced through microbial fermentation, presents an innovative approach to enhancing skin health while addressing environmental concerns. Primarily, FC was enzymatically hydrolyzed by Prozyme 2000P peptidase (1% enzyme-add, 10% substrate, pH 7.0, 50 °C, and 12 h reaction) (Figure 1A). The low-molecularized FC, with added raw materials (1% L-glutamic acid, 1% yeast extract, 0.5% hydrated glucose, and water), was sequentially fermented twice by two types of lactobacillus to produce GBL extract. Finally, the product labeled as ‘MBP-GABALAGEN-20230516’, was powdered and used in pre-clinical and clinical randomized studies in the form of a stick jelly formula to investigate its biological potential for skin health (Figure 1A).

To recognize a key indicating component in GBL, HPLC analysis was applied (Figure 1B). These stacked comparison chromatograms showed that a distinct level of GABA substance is present in GBL compared to the reference material and blanks (Figure 1B). Therefore, as GBL was clearly shown to contain GABA, a concentration-dependent reference material standard calibration curve was prepared under the same analytical conditions to accurately quantify the GABA content (y=21.2720x+8.0540) (Figure 1C). Based on the standard calibration curve, we evaluated the GABA content produced during the sequential lactic acid bacteria fermentation process of low-molecular-weight fish collagen by assessing the amount of GABA generated over different fermentation time periods (Figure 1D). As a result, the highest GABA production rate (10.08%) was observed at 48 h fermentation (24 h and 24 h) after primary inoculation during the two-stage sequential lactobacillus culture process (fermentation 1st and 2nd) (Figure 1A,D). In conclusion, GBL produced under optimal microbial fermentation conditions for 48 h from low-molecular-weight-fish-scale-derived FC was verified to contain approximately 9.955 ± 0.08–9.958 ± 0.10% of GABA, regardless of the sample amount used for HPLC assessment (Figure 1E).

Next, to elucidate the nutritional value of GBL, the content of free amino acids (FAAs) in the material was evaluated. GBL exhibited an FAA content of 22,070 mg/100 g, which was approximately 16.47-fold higher than that of FC, the raw material used in GBL production (Figure 1F). Among the 18 identified FAAs in GBL, GABA accounted for the highest proportion at 38.83% of the total FAA content, followed by glutamine (Glu) at 13.55%, which was the second highest in GBL (Figure 1G). In summary, this research successfully transformed marine collagen derived from fishery byproducts into a high-value biomaterial containing high concentrations of GABA through microbial fermentation processes. Our study emphasized the nutritional value of GBL, which demonstrates superior bioavailability compared to conventional collagen materials, based on its rich FAA content.

### 3.2. GBL Supplementation Safely Mitigates Aging Dermal Health in Human Randomized Trial

**Figure 2:**
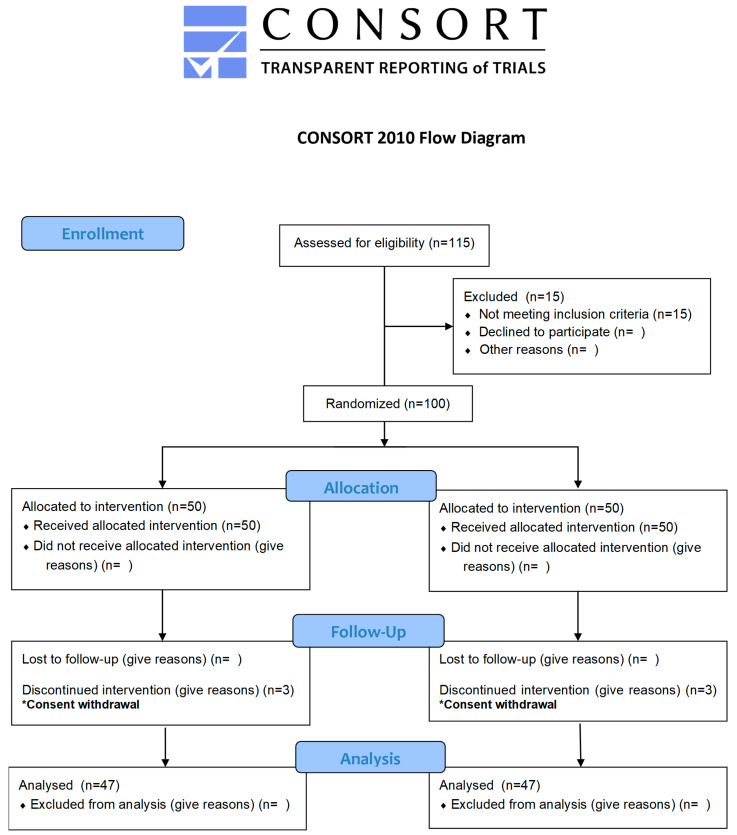
The regular Consolidated standards of reporting trials (CONSORT) 2010 flow diagram for patient selection.

**Figure 3:**
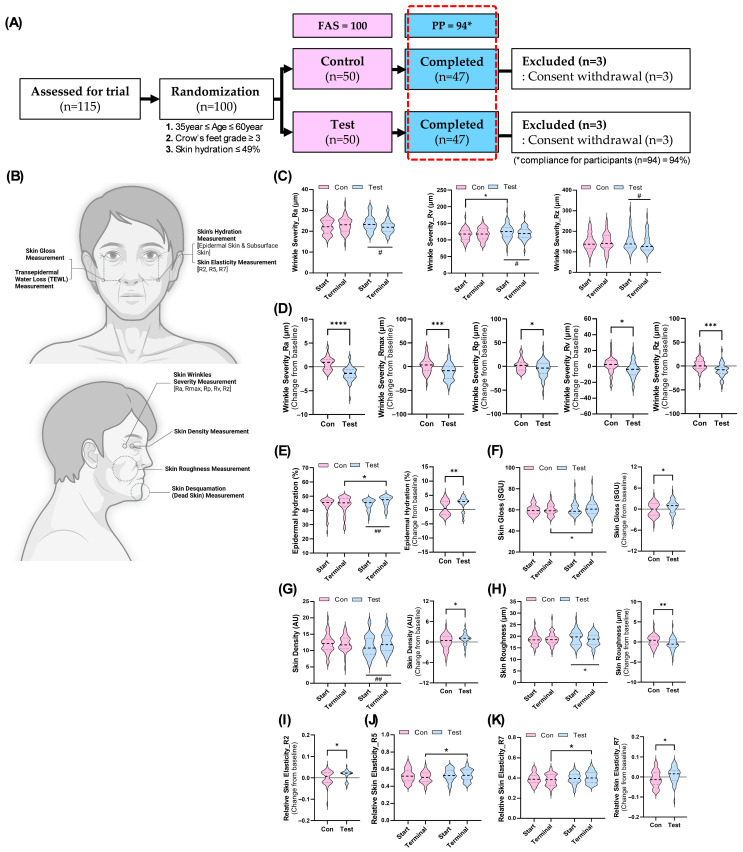
The regular GBL treatment protects skin aging in human randomized trials. (**A**) The randomized study scheme designed for control and test groups, and the number of participants excluded in each group for reasons indicated. (**B**) Assessment diagram of facial areas for skin health evaluations by analytical techniques. (**C**) The comparison measurement of average wrinkle severity (Ra, Rv, and Rz) between the groups before and after sample intake. (**D**) The changes from baseline of wrinkle severity in GBL-supplemented test and control groups. (**E**) The comparison measurement of average epidermal hydration rates between the groups before and after sample intake, as well as the changes in epidermal hydration rates from baseline for each group. (**F**) The comparison measurement of average skin gloss rates between the groups before and after sample intake, as well as the changes in skin gloss rates from baseline for each group. (**G**) The comparison measurement of average skin density rates between the groups before and after sample intake, as well as the changes in skin density rates from baseline for each group. (**H**) The comparison measurement of average skin roughness rates between the groups before and after sample intake, as well as the changes in skin roughness rates from baseline for each group. (**I**) The changes from baseline of relative skin elasticity (R2) in GBL-supplemented test and control groups. (**J**) The comparison measurement of relative skin elasticity (R5) between the groups before and after sample intake. (**K**) The comparison measurement of average relative skin elasticity (R7) rates between the groups before and after sample intake, as well as the changes in relative skin elasticity (R7) rates from baseline for each group. Data are shown to mean ± S.D. (*n* = 47). *^,#^ *p* < 0.05, **^,##^ *p* < 0.01, *** *p* < 0.001, and **** *p* < 0.0001.

This randomized, double-blind, placebo-controlled trial aimed to evaluate the efficacy and safety of GBL supplementation in improving age-related skin damage in adults aged 35 to 60 years old. Those who did not meet the inclusion criteria (*n =* 15) were excluded from the assessed eligibility (*n =* 115) (Figure 2). A total of 100 participants with significant skin aging concerns, such as crow’s feet wrinkles graded at level 3 or higher and skin moisture levels below 49%, were finally enrolled (Figure 2). A total of 6 participants dropped out during the study, leaving 94 participants who completed the trial, resulting in a compliance rate of 94% (Figure 2 and Figure 3A). The FAS included all 100 participants (50 per group), while the PP analysis included the 94 participants who completed the trial (47 per group), to assess GBL’s impact on skin health (Figure 3A).

Baseline characteristics, including age, height, weight, cardiovascular conditions, skin condition, gender, smoking status, and drinking status, showed no significant differences between the test and control groups in either the FAS or PP populations (Table 2). Nutritional intake parameters such as total calorie consumption, protein intake, and water intake were monitored throughout the trial from Week 0 (‘Start’) to Week 12 (‘Terminal’) (Table 3). No notable changes were observed in these parameters between the two groups during the study period (Table 3). Safety assessments revealed that while γ-GTP levels in the test group were slightly lower at baseline compared to the control group, no abnormal findings were detected among individual subjects (Table S3). All safety-related factors remained within normal ranges throughout the trial.

Efficacy evaluations focused on various skin health parameters, including hydration, wrinkle severity, elasticity, density, roughness, glossiness, and desquamation. These indicators were measured across specific facial areas using advanced imaging and analytical techniques (Figure 3B). These dermal health-related indicators were then compared to determine whether GBL supplementation had a statistically significant impact on skin health compared to the control group. The influence of GBL supplementation on wrinkle severity was assessed using a 3D optical imaging device based on fringe projection technology. Key wrinkle parameters analyzed included Ra (average roughness), Rmax (maximum peak-to-valley distance), Rp (highest peak height), Rv (wrinkle depth), and Rz (average peak-to-valley distance) (Figure 3C,H). Regular GBL consumption demonstrated significant improvements in wrinkle severity, particularly in the crow’s feet area. While no significant differences were observed between groups for most indices except Rv at baseline, within-group comparisons revealed that GBL intake led to a marked reduction in wrinkle depth (Rv) and average roughness (Ra) (Figure 3C). The Rz index also showed significant improvement in the test group (Figure 3C). However, no changes were observed for Rmax or Rp (Figure S1A,B). Further analysis showed significant improvements within the test group across all five wrinkle indicators when comparing changes from baseline to post-GBL intervention (Figure 3D). Additionally, GBL supplementation significantly improved other skin health indicators such as epidermal hydration, density, roughness, and glossiness (Figure 3E–H). Notably, except for skin glossiness, these improvements were statistically significant at the end of the intake period within the test group (Figure 3E,G,H).

Skin elasticity was also assessed through gross elasticity (R2), net elasticity (R5), and biological elasticity (R7) (Figure 3I–K). Regular GBL intake resulted in significant increases in R2 and R7 from baseline (Figure 3I,J). At the end of the trial, between-group comparisons indicated that R5 and R7 values were significantly higher in the test group compared to controls (Figure 3J,K). These findings suggest that GBL supplementation may enhance skin elasticity. However, other measured indicators such as subsurface hydration, desquamation levels, and TEWL did not show significant improvements with GBL consumption (Figure S1C–E). In conclusion, cumulative intake of GBL demonstrated its potential to alleviate various signs of age-related skin deterioration by improving parameters such as wrinkle severity, hydration levels, density, roughness, and elasticity. These findings indicate that GBL supplementation may serve as an effective intervention for enhancing overall skin health in aging adults.

### 3.3. GBL Treatment Restore UVB-Irradiated Oxidative Stress in Human Skin Cells

**Figure 4:**
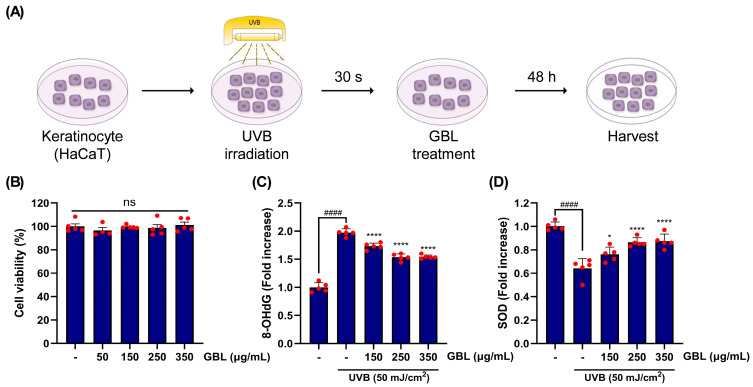
The protective effect of GBL against UVB-stimulated photo-irradiation on human keratinocytes. (**A**) The diagram of experimental procedure for the potential role of GBL in UVB-irradiated HaCaT cells. (**B**) The cytotoxicity of GBL treatment with the indicated range of concentrations. (**C**) Evaluation of GBL’s protective effects on UVB-stimulated HaCaT cells through measurement of 8-OHdG secretion. (**D**) Evaluation of GBL’s protective effects on UVB-stimulated HaCaT cells through measurement of SOD secretion. Data are displayed to mean ± S.D. (*n* = 5). ns; not significant. * p < 0.05 and **** *p* < 0.0001 vs. UVB-only-stimulated blank group. ^####^ *p* < 0.0001 vs. control group.

Upon trial, GBL application significantly improved aging-linked dermal health phenotypes. GBL was evaluated to determine whether it protects against UVB-driven oxidative stress in HaCaT human keratinocytes. To investigate this, GBL was supplemented post-UVB irradiation and tested after 48 h of incubation (Figure 4A). A range of GBL treatment concentrations (350–50 μg/mL) did not exhibit cytotoxicity in human keratinocytes; however, the group treated with 50 μg/mL of GBL displayed relatively lower cell viability compared to others. Therefore, the 150–350 μg/mL range was selected for further experiments (Figure 4B). UVB stimulation notably elevated the levels of 8-OHdG, a marker of oxidative-stress-induced DNA damage, but this increase was statistically mitigated by GBL treatment in a dose-dependent manner (Figure 4C). In addition, UVB exposure distinctly downregulated SOD, an enzyme with an antioxidant role under oxidative stress conditions (Figure 4D). However, this oxidative stress-relieving mechanism was significantly restored by dose-dependent GBL treatment (Figure 4C). Taken together, these results suggest that oxidative stress and damage to skin cells caused by UVB occur through various mechanisms. However, these effects are inhibited by GBL supplementation, which enhances antioxidant enzyme activity and promotes DNA repair.

### 3.4. GBL Treatment Improves Gene Expressions Related to Dermal Damage Caused by UVB Exposure

**Figure 5:**
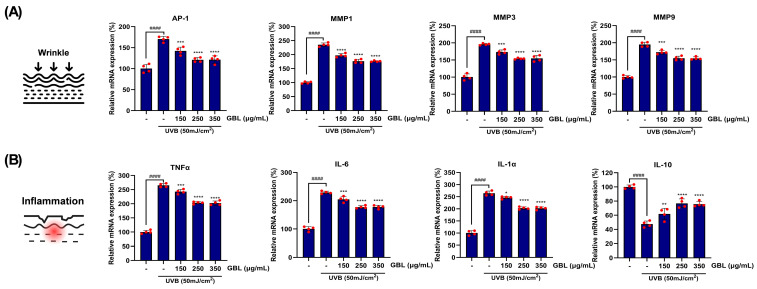
Quantitative evaluations of the impacts of GBL treatment on the gene expressions associated with UVB stress in human keratinocytes. (**A**) Relative mRNA gene expressions regulated by the dose-dependent GBL exposure under UVB stimulation, linked to wrinkle formation. (**B**) Relative mRNA gene expressions regulated by the dose-dependent GBL application under UVB stimulation, linked to skin inflammation. Data are depicted to mean ± S.D. (n = 4). * *p* < 0.05, ** *p* < 0.01, *** *p* < 0.001, and **** *p* < 0.0001 vs. only-UVB-stimulated blank group. ^####^ *p* < 0.0001 vs. control group.

The restorative effect of GBL application on skin cells was also demonstrated at the gene expression level (Figure 5). Significant UVB irradiation (50 mJ/cm^2^) on HaCaT cells markedly increased the expression of the UV-induced transcription factor AP-1 gene by 170.45%. Additionally, the expression of target genes related to dermal collagen degradation increased by 235.26%, 195.84%, and 194.87% for metalloproteinase (MMP)-1, MMP3, and MMP9, respectively, compared to the control group (Figure 5A). However, when GBL was applied to UVB-stimulated HaCaT cells in a concentration-dependent manner (150–350 μg/mL), the expression of these UVB-induced AP-1 downstream genes was significantly reduced compared to the control group. In the group treated with the highest concentration of GBL, the reduction rates were 25.39%, 24.70%, and 19.08%, respectively (Figure 5A).

Among the target genes of AP-1 induced by UVB exposure, the major pro-inflammatory cytokines TNF-α, IL-6, and IL-1α were regulated in a dose-dependent manner by GBL treatment in human keratinocytes (Figure 5B). At the highest concentration, the maximum inhibitory effects compared to the control group were observed as 21.22%, 22.49%, and 19.67%, respectively, suggesting that GBL treatment alleviates inflammatory responses (Figure 5B). Furthermore, the suppression of IL-10 (an immunosuppressive regulatory cytokine) gene expression, which is activated as a negative feedback mechanism of UVB radiation, was observed (Figure 5B). This immunosuppressive mechanism was significantly improved by high-dose GBL treatment, resulting in a 37.90% increase, and this activation was found to occur in a dose-dependent manner (Figure 5B).

Exposure to GBL was found to significantly alleviate oxidative-stress-induced cell death (including collagenase activation and inflammatory responses) caused by UVB stimulation, a major environmental factor contributing to aging, in human skin cells. These findings suggest that the activation of oxidative stress defense mechanisms played a key role in the significant improvements observed in aging-related skin health indicators at the human level following GBL consumption.

## 4. Discussion

GBL, developed by combining low-molecular-weight collagen derived from fish scales with GABA through lactobacillus fermentation, represents a significant advancement in nutricosmetics [8]. Compared to traditional collagen-based products, GBL offers enhanced benefits for skin health through its synergistic composition and innovative production process in our previous study [14]. Traditional marine collagen-based nutricosmetics, such as hydrolyzed FC peptides, have been shown to improve skin hydration, elasticity, and wrinkle reduction [19]. Randomized trials have demonstrated that fish-derived low-molecular-weight collagen (LMWC), due to its smaller molecular size, is absorbed more efficiently into the bloodstream and dermis, enhancing its bioavailability and efficacy. Several studies have reported significant improvements in skin hydration and elasticity after 12 weeks of LMWC supplementation, with reductions in wrinkle depth and skin roughness [20,21]. Also, marine-derived collagen peptides have been particularly praised for their superior bioavailability compared to land-based sources [22].

Despite its proven benefits for skin health, there are several hurdles that hinder the industrial application of LMWC. One significant limitation is its thermal stability. FC has a lower denaturation temperature compared to mammalian collagen, which makes it less suitable for applications that require high thermal stability, such as medical-grade products [23,24]. Another challenge lies in its oxidative stability. Fish collagen is susceptible to oxidative degradation during processing and storage, which can negatively affect the quality of the final product [25]. Therefore, in the industrial setting, these limitations that shorten the shelf life of FC are ultimately cited as the primary factor driving up overall product costs, and there is growing demand for improvements. Interestingly, several studies indicate that lactobacillus fermentation can extend the shelf life of protein-based products [26,27]. Lactobacillus fermentation achieves this by creating an acidic environment, producing antimicrobial compounds, and modifying protein structures, which collectively inhibit spoilage and pathogenic microorganisms, and fermentation further reduces the molecular weight of collagen peptides, enhancing their absorption by the body [15,28]. Therefore, to apply the benefits conferred by fermentation, we attempted GBL production via bioconversion through LMWC fermentation (Figure 1A).

Furthermore, we were able to anticipate additional improvements in stability and the introduction of functional properties through the generation of GABA during the lactobacillus fermentation process. Studies have shown that GABA exhibits significant antioxidant properties. For instance, during fresh cheese fermentation, GABA production was associated with increased ABTS antioxidant and metal-chelating activities, indicating its potential to reduce oxidative stress in food products [29]. As shown in Figure 1, GBL was found to produce a significant amount of GABA during the production process, with GABA being the most abundant FAA contained within GBL (Figure 1D,G). These facts indicate that GABA’s inclusion in GBL introduces unique benefits that traditional collagen products lack. According to a variety of previous reports, GABA upregulates type I collagen expression in dermal fibroblasts, promoting skin elasticity and reducing wrinkles [30]. And GABA treatment not only significantly inhibited UVB-induced matrix MMP-1, which degrades collagen, but also improved skin hydration by expression of aquaporin-3 in keratinocytes [31].

The efficacy of GBL was validated through a randomized, double-blind, placebo-controlled trial involving 100 participants (aged 35–60 years) with visible signs of aging (Figure 2 and Figure 3A). While traditional collagen supplements primarily target hydration and elasticity, GBL’s unique combination of LMWC and GABA widely offers significant improvements in multiple skin health parameters, including hydration, elasticity, wrinkle reduction, and skin density, without adverse effects during the study period, confirming its safety for long-term use (Figure 3 and Table S3). Supplementation of GBL significantly showed a skin improvement in hydration (increase in 20%) and wrinkle depth (decrease in 15%) after 12 weeks (Figure 3C–E). In addition, GBL consumption notably enhanced skin density and reduced roughness and significantly upregulated the elasticity-indicating parameters (R2, R5, and R7), outperforming traditional collagen formulation (Figure 3G,H).

The in vitro experiments using UVB-irradiated HaCaT keratinocytes further elucidated the molecular mechanisms underlying these clinical effects (Figure 4 and Figure 5). GBL treatment restored oxidative stress markers such as 8-OHdG and SOD levels in a dose-dependent manner (Figure 4). This suggests that GBL mitigates UVB-induced oxidative stress through its potent antioxidant properties. Additionally, GBL reduced the gene expression of pro-inflammatory cytokines and MMPs, which are known to degrade collagen and exacerbate skin aging (Figure 5). The clinical improvements observed in skin hydration, elasticity, and wrinkle depth can be attributed to GBL’s dual action at the cellular level: the UVB-linked oxidative stress and antioxidant pathway mechanism in human skin. These effects are attributed to GBL’s ability to combat UVB-induced oxidative stress in human keratinocytes, reduce pro-inflammatory cytokine expression, and enhance antioxidant enzyme activity (Figure 3, Figure 4 and Figure 5).

GBL maximizes functionality and not only increases the added value of FC, which uses fish scale byproducts from the fishery industry as its main raw material but also proposes a new solution for improving stability—a weakness of seafood—thereby contributing to the revitalization of related industries and aligning with global sustainability trends. Additionally, the results of this study, which produce GABA through the curated lactobacillus fermentation, can overcome the limitations of traditionally recognized functional materials with established safety and provide novelty in bioavailability [32]. This can be applied to enhance the value of various materials. Such an upcycling approach not only reduces cost inefficiencies and environmental impacts associated with the development of new materials but also supports the principles of a circular economy.

While the randomized trial demonstrated GBL’s efficacy in improving skin health, limitations include the lack of subgroup analyses based on age and gender. These factors may influence treatment responses due to physiological differences, such as collagen metabolism and hormonal variations. Future studies should stratify participants by these demographics to assess differential effects, ensuring a comprehensive understanding of GBL’s efficacy across diverse populations. For instance, populations should be studied in the variable types of skin hydration, wrinkle depth, and elasticity separately for men and women to identify potential sex-specific responses. Similarly, different skin types may need different doses of GBL to see the desired effects.

## 5. Conclusions

This study highlights the development and evaluation of GBL, a novel nutricosmetic ingredient combining low-molecular-weight fish collagen and GABA, produced through sustainable microbial fermentation. GBL demonstrated significant improvements in skin health, including enhanced hydration, elasticity, wrinkle reduction, and skin density, as validated through a randomized trial. These effects were achieved without adverse events, confirming the safety of GBL consumption for long-term use. Additionally, GBL exhibited protective properties against UVB-induced oxidative stress and inflammation in human keratinocytes, underscoring its multifunctional benefits. By upcycling marine byproducts into high-value biomaterials, GBL aligns with global sustainability goals and offers an innovative solution for addressing age-related skin concerns while promoting environmental responsibility. Future research should explore its efficacy across diverse populations to optimize its broader application.

## Figures and Tables

**Table 1:** List of primers for qRT-PCR.

Gene		Primers
*Actb* (Gene ID: 60)	*Forward*	5′-*CTGGAACGGTGAAGGTGACA*-3′
	*Reverse*	5′-*AAGGGACTTCCTGTAACAATGCA*-3′
*Tnf*-*a* (Gene ID: 7124)	*Forward*	5′-*CCTCTCTCTAATCAGCCCTCTG*-3′
	*Reverse*	5′-*GAGGACCTGGGAGTAGATGAG*-3′
*Il*-1*a* (Gene ID: 3552)	*Forward*	5′-*GGCATCACTGTTGCTTCGG*-3′
	*Reverse*	5′-*GCCAGTCTAATTCTCCTGGTCA*-3′
*Il*-6 (Gene ID: 3569)	*Forward*	5′-*ACTCACCTCTTCAGAACGAATTG*-3′
	*Reverse*	5′-*CCATCTTTGGAAGGTTCAGGTTG*-3′
*IL*10 (Gene ID: 3586)	*Forward*	5′-*GCCTAACATGCTTCGAGATC*-3′
	*Reverse*	5′-*TGATGTCTGGGTCTTGGTTC*-3′
*AP*-1 (Gene ID: 3725)	*Forward*	5′-*ACTCATACACAGCTACGGGATACG*-3′
	*Reverse*	5′-*GGGTCGGCCAGGTTGAC*-3′
*MMP*1 (Gene ID: 4312)	*Forward*	5′-*ACATTGCAGGATGTGCAGGCTCTT*-3′
	*Reverse*	5′-*CTTGGGTACTGGTGACCGGTGTCA*-3′
*MMP*3 (Gene ID: 4314)	*Forward*	5′-*AGC TGA GTA CCG AGA GAT CGA C*-3′
	*Reverse*	5′-*TCA GCC ACA TCA AGT ATT GGT C*-3′
*MMP*9 (Gene ID: 4318)	*Forward*	5′-*GAC ATG CCC AAG ACT CAG AAG T*-3′
	*Reverse*	5′-*ACT TCC TTT CCT TCT CCT TTG C*-3′

**Table 2:** Characteristics of the participants in the current randomized trial.

Variable		FAS Population			PP Population		
		Control (*n* = 50)	Test (*n* = 50)	*p*-Value **	Control (*n* = 47)	Test (*n* = 47)	*p*-Value **
Age (years)		47.76 ± 6.10	47.82 ± 6.18	0.961 ^1)^	47.83 ± 6.11	48.00 ± 6.26	0.894 ^1)^
Height (cm)		164.01 ± 8.47	163.35 ± 8.31	0.692 ^1)^	163.94 ± 8.67	163.16 ± 8.22	0.653 ^1)^
Weight (kg)		63.11 ± 10.26	59.59 ± 10.56	0.094 ^1)^	62.89 ± 10.45	59.19 ± 9.82	0.080 ^1)^
Diastolic BP (mmHg)		71.74 ± 14.67	69.04 ± 11.06	0.301 ^1)^	71.81 ± 14.83	69.17 ± 11.07	0.331 ^1)^
Systolic BP (mmHg)		119.52 ± 18.62	118.14 ± 14.39	0.679 ^1)^	119.45 ± 18.84	118.26 ± 14.52	0.732 ^1)^
Pulse rate		76.32 ± 10.20	77.00 ± 8.63	0.720 ^1)^	76.15 ± 10.49	77.23 ± 8.62	0.585 ^1)^
Skin Hydration		43.55 ± 6.35	44.50 ± 3.79	0.367 ^1)^	43.25 ± 6.43	44.39 ± 3.88	0.302 ^1)^
Sex	Male	12 (24.0%)	13 (26.0%)	0.817 ^2)^	11 (23.4%)	12 (25.5%)	0.810 ^2)^
	Female	38 (76.0%)	37 (74.0%)		36 (76.6%)	35 (74.5%)	
Smoker	None	46 (92.0%)	46 (92.0%)	1.000 ^3)^	44 (93.6%)	44 (93.6%)	1.000 ^3)^
	Ex	1 (2.0%)	1 (2.0%)		1 (2.1%)	1 (2.1%)	
	Current	3 (6.0%)	3 (6.0%)		2 (4.3%)	2 (4.3%)	
Drinker	None	17 (34.0%)	24 (48.0%)	0.155 ^2)^	17 (36.2%)	23 (48.9%)	0.211 ^2)^
	Moderate	33 (66.0%)	26 (52.0%)		30 (63.8%)	24 (51.1%)	
	Heavy	0 (0.0%)	0 (0.0%)		50(0.0%)	0 (0.0%)	

** *p*-values were compared between groups; ^1)^ independent *t*-test; ^2)^ Chi-square test; ^3)^ Fisher’s exact test.

**Table 3:** The caloric and nutritional consumption by the study groups.

Variable	Observed Value (PP)			Change from Baseline				
	Control (*n* = 47)	Test (*n* = 47)	*p*-Value **	Control	*p*-Value *	Test	*p*-Value *	*p*-Value **
Total calorie consumption (Kcal)								
Start	1331 ± 395	1252 ± 342	0.306 ^1)^					
Terminal	1323 ± 337	1303 ± 333	0.771 ^1)^	−8. ± 369	0.884 ^2)^	60 ± 303	0.259 ^2)^	0.404 ^1)^
Protein intake (g)								
Start	53.56 ± 15.62	50.20 ± 12.38	0.250 ^1)^					
Terminal	53.12 ± 14.04	51.70 ± 12.27	0.603 ^1)^	−0.44 ± 18.84	0.873 ^2)^	1.50 ± 15.70	0.515 ^2)^	0.588 ^1)^
Water intake (mL)								
Start	612.0 ± 174.3	562.4 ± 158.6	0.152 ^1)^					
Terminal	590.0 ± 161.8	598.3 ± 145.3	0.794 ^1)^	−22.0 ± 191.2	0.434 ^2)^	35.9 ± 218.2	0.265 ^2)^	0.174 ^1)^

* *p*-values were compared within each group; ** *p*-values were compared between groups; ^1)^ Independent *t*-test; ^2)^ paired *t*-test.

## Data Availability

Data are contained within this article.

## Supplementary Materials

The following supporting information can be downloaded at: https://www.mdpi.com/article/10.3390/antiox14030245/s1, Figure S1: Measurement of additional skin health indicators following GL administration in human clinical trial; Table S1: Detailed exclusion criteria for clinical study; Table S2: The test and placebo ingredient’s formular for the clinical trial; Table S3: The clinical safety test results in participants’ serum in between test and control groups.
