# Supplementary material for: Targeting Aging Skin with GABALAGEN®: A Synergistic Marine Nutricosmetic Ingredient Validated Through Human Randomized Trials
Source: Antioxidants (Basel). 2025 Feb 20;14(3):245. doi: 10.3390/antiox14030245 (PMC11939556; doi:10.3390/antiox14030245)
Supplement: Supplementary file 1 [file antioxidants-14-00245-s001.zip › antioxidants-3476487-supplementary.pdf]

Supplementary Materials and Methods (Tables)

Supplementary methods

1. The total calorie intake, protein intake, and water intake based on the 24-h recall method

1.1. Total Calorie Intake Assessment

Assessment of the total energy consumed by an individual within a 24-h period, recorded in kilocalories (kcal), using dietary recall data.

1.2. Protein Intake Assessment

Measurement of the total protein consumed (in grams) over a 24-h period, derived from self-reported food and beverage consumption data.

1.3. Water Intake Evaluation

Evaluation of the total volume of water consumed (in mL) within a 24-h timeframe, including water from beverages and food sources.

2. Evaluation Criteria, Methods, and Reporting of Safety, Including Adverse Reactions

2.1. Subjects of Safety Evaluation and Evaluation Methods

The subjects for the safety evaluation include all individuals who were randomly assigned to the study. Adverse reactions are assessed based on their severity, categorized into three levels: mild, moderate, and severe. The causal relationship between test food and the adverse reaction is evaluated on a six-point scale. If abnormal changes are detected in clinical laboratory test results or vital signs, specific symptoms are identified, and their clinical significance is assessed.

2.2. Classification and Definition of Adverse Reactions

An adverse reaction refers to any event that occurs during the observation period of a human application trial that negatively affects the well-being of the study participant, including diseases and accidents. This also encompasses clinically significant laboratory test results or diagnostic findings unrelated to the primary condition being studied. Examples include events that necessitate unplanned diagnostic procedures or treatment or cause participants to withdraw from the study.

- Adverse Event (AE): This refers to undesirable or unintended symptoms, signs, or diseases occurring after consuming the test food in a human application trial. A causal relationship with the test food is not necessarily required.

- Adverse Drug Reaction (ADR): This is an adverse event for which a causal relationship with the test food cannot be completely ruled out.

- Serious Adverse Event/Reaction (SAE): This includes adverse events or reactions that result in death, are life-threatening, require hospitalization or prolonged hospitalization, cause persistent or significant disability or impairment, lead to congenital anomalies or birth defects, or are otherwise medically significant.

2.3. Collection and Documentation of Adverse Reactions

Adverse reactions are collected from the time of test food consumption until the final visit during the human application trial period. Any medical events occurring before consumption of the test food are recorded as current medical history. Reports on adverse reactions must include details such as the name of the reaction, duration (start and end dates), severity, causal relationship with the test food, outcomes, corrective treatments taken, and whether it qualifies as a serious adverse reaction. When documenting adverse reactions, investigators should use standardized medical terminology to summarize diagnoses or symptoms rather than listing individual signs or symptoms. Adverse reactions occurring during the trial should be monitored until they resolve, stabilize with a definitive outcome, or until follow-up becomes impossible. Adverse reactions occurring after the trial ends should only be reported if they are serious and related to the test food.

2.4. Evaluation of Adverse Reactions

2.4.1. Evaluation Criteria

Adverse reactions are classified into three levels for evaluation—Mild: No interference with daily activities; Moderate: Interferes with daily activities and requires simple treatment; Severe: Prevents daily activities and requires intensive treatment.

2.4.2. Causality Assessment

The causal relationship between the test food and an adverse reaction is categorized as follows—Clearly related; Probably related; Possibly related; Probably not related; Clearly not related; Unknown.

2.5. Reporting Serious Adverse Reactions

All serious adverse reactions occurring during the human application trial must be reported to the sponsor within 24 hours of recognition by the investigator, regardless of their relationship to the test food or its consumption status. Reports can be made via phone or written documentation. To protect participant confidentiality, identifying information such as name, identification number, and address should be replaced with a participant identification code in all reports. If necessary medical records are submitted as part of reporting, identifying information must also be redacted except for identification codes. Investigators must provide follow-up reports if additional information on serious adverse reactions becomes available and must continue reporting periodically until these reactions are resolved (e.g., disappearance of symptoms or inability to follow up).

3. Ethical Considerations and Administrative Procedures

3.1. Compliance with the Human Application Study Protocol

Researchers must conduct human application studies in strict compliance with the approved study protocol. Deviations from the protocol are not permitted unless immediate action is required to eliminate risks to participants. In cases of protocol violations, the details and reasons must be documented. Even if a researcher believes that deviations could improve the study, no changes may be implemented without prior agreement from the sponsor and approval from the Institutional Review Board (IRB).

3.2. Approval and Modification of the Study Protocol

To initiate or modify a human application study, researchers must obtain IRB approval for the study protocol or any proposed amendments.

3.3. Informed Consent Process for Participants

The principal investigator and study personnel must provide potential participants with clear explanations of the study's purpose, procedures, potential health risks, and compensation policies before obtaining written consent. Explanations may be given both verbally and in writing, and sufficient opportunities must be provided for participants or their legal representatives to ask questions about the study.

3.4. Measures for Participant Safety and Protection

Researchers are required to conduct studies in accordance with the Declaration of Helsinki, prioritizing participant rights and welfare. All personnel involved in the study must be well-versed in the protocol. Researchers should allocate sufficient time to evaluate participant eligibility and monitor for adverse events through interviews and examinations. The principal investigator must regularly report adverse events, progress, circumstances, and outcomes to the sponsor, who will oversee the study's progress.

3.5. Compensation for Participants

The sponsor is responsible for compensating participants for injuries directly caused by the investigational product or during corrective treatment for adverse events, in accordance with established compensation policies.

3.6. Documentation and Record Keeping

3.6.1. Case Report Forms (CRFs) and Source Documents

Data collected during the study will be recorded using CRFs based on source documents. All entries in CRFs must be accurate, complete, legible, and timely. Any changes or corrections should retain visibility of the original content. Monitors will compare CRFs with source documents to identify discrepancies and

request appropriate corrections from researchers.

3.6.2. Access to Source Documents

Sponsors, monitors, and auditors may access participant records for monitoring, auditing, or study management purposes. Researchers must ensure that such access complies with confidentiality standards and that facilities meet security requirements.

3.6.3. Storage of Study Materials

Researchers must securely store all materials related to the study for at least three years after its completion or termination. After finalizing reports, documents should be transferred to a designated custodian. Any plans to destroy or relocate records must be communicated to the sponsor in advance.

3.7. Confidentiality of Study Documents and Participant Records

All results and documents from human application studies are treated as confidential. Participant-identifiable data will be anonymized using identification codes instead of names. Even if results are published, participant identities will remain confidential.

3.8. Monitoring by Study Sites

The sponsor will conduct monitoring visits to ensure compliance with the approved protocols and assess participant safety and human rights protection. Monitors will review CRFs, source documents, and supplementary materials while engaging with investigators when necessary. The sponsor appoints at least one monitor to oversee study progress at each site.

3.9. Suspension of Human Application Studies

If a site fails to comply with protocols or contractual agreements, the sponsor will take corrective action immediately. Persistent non-compliance or issues such as failure to meet enrollment targets or significant safety concerns may result in suspension of the site’s participation in the study.

3.10. Reporting and Publication of Study Results

Once data from all participating sites have been fully analyzed, the sponsor will prepare a report summarizing the findings for investigators. The sponsor retains ownership of all data and results from the study and has exclusive rights to publish them. Investigators must not publish or disclose results without prior written consent from the sponsor. Drafts of any publications or presentations must be reviewed by the sponsor before dissemination to ensure accuracy and approval.

Table legends

Table. S1. Detailed exclusion criteria for clinical study.

Table. S2. The test and placebo ingredient's formular for the clinical trial.

The group of essential amino acid (EAA) represents to the sum of each amino acid, including arginine, histidine, iso-leucine, lysine, methionine, phenylalanine, threonine, tryptophane, and valine. The group of branched chain amino acids represents to the sum of valine, iso-leucine, and leucine.

Table. S3. The clinical safety test results in participants' serum in between test and control groups. Steps 1–7 were performed by MSBIO Co. Ltd. (HACCP approval no.: 2022-2-0656, South Korea). Steps 8–9 were performed by S&D Co., Ltd. (GMP approval no.: 20130011, Cheongju, Chungcheongbuk-do, South Korea). The entire mass production process was performed between October 2022 and November 2023.

Table S1 – The detailed exclusion criteria for clinical study

**Excluded criteria**	**1. Individuals with the following conditions:**<br>① Uncontrolled hypertension (systolic blood pressure ≥ 160 mmHg or diastolic blood pressure ≥ 100 mmHg).<br>② Diabetes requiring antidiabetic medication (oral hypoglycemic agents, insulin, etc.) or fasting blood glucose levels of 126 mg/dL or higher.<br>③ Liver dysfunction (AST or ALT ≥ 120 U/L) or hepatobiliary diseases.<br>④ Renal dysfunction (creatinine levels ≥ 2.6 mg/dL).<br>**2. Individuals who have been continuously consuming health supplements (collagen, ceramide, hyaluronic acid) or herbal medicine that may affect the skin within one month prior to the first visit.**<br>**3. Individuals who have been continuously taking antibiotics within two weeks prior to the first visit or are likely to take them during the study period.**<br>**4. Pregnant or breastfeeding individuals, or those planning to become pregnant.**<br>**5. Individuals with irritation or allergies to cosmetic, pharmaceutical, or test sample-related ingredients in food products.**<br>**6. Individuals who have used topical steroids or taken oral retinoids/steroids within six months prior to the start of the study.**<br>**7. Individuals who have used functional cosmetics or highly moisturizing cosmetics within two weeks prior to the start of the study.**<br>**8. Individuals who have undergone or plan to undergo procedures (chemical peeling, Botox, other skin treatments, etc.) on the test area within three months prior to the start of the study.**<br>**9. Individuals who have participated in a similar study within the past three months.**<br>**10. Individuals with diseases that may affect the study (e.g., heart, kidney, liver, thyroid, cerebrovascular diseases, gallbladder diseases, gastrointestinal disorders, gout).**<br>**11. Individuals with skin conditions (e.g., atopic dermatitis) on the test area.**<br>**12. Individuals with chronic wasting diseases (e.g., asthma, diabetes, hypertension).**<br>**13. Individuals with mental illnesses (e.g., depression, schizophrenia, alcoholism, drug addiction).**<br>**14. Individuals taking obesity treatments (absorption inhibitors, antidepressants, appetite suppressants), contraceptives, hormonal agents, or diuretics.**<br>**15. Individuals consuming excessive alcohol (average daily intake of 30 g or more).**<br>**16. Individuals with sensitive or hypersensitive skin.**<br>**17. Individuals with skin abnormalities such as moles, acne, erythema, or telangiectasia on the test area.**<br>**18. Any other cases deemed unsuitable for participation in the study by the principal investigator's judgment.**

Table S2. Test formular table

	Placebo formular			Test formular		
**Purpose**	**Ingredient Name**	**Proportion (%)**	**Among (mg)**	**Ingredient Name**	**Proportion (%)**	**Among (mg)**
**Main Ingredient**				**Gabalagen (GL)**	**7.5**	**1,500**
	Fructo-oligosaccharide (F250)	10	2,000	Fructo-oligosaccharide (F250)	10	2,000
	Crystalline Fructose	8	1,600	Crystalline Fructose	8	1,600
	Foodgel A	2.1	420	Foodgel A	2.1	420
	Orange Flavor (FN0112-0001)	1.6	320	Orange Flavor (FN0112-0001)	1.6	320
	Citric Acid	0.6	120	Citric Acid	0.6	120
**Bulking ingredients** 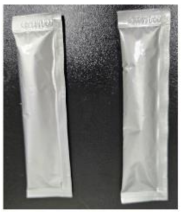 **Placebo Test**	Orange Extract	0.4	80	Orange Extract	0.4	80
	Enzyme-Treated Stevia	0.35	70	Enzyme-Treated Stevia	0.35	70
	Calcium Lactate	0.3	60	Calcium Lactate	0.3	60
	Carrageenan Blend Additive	0.2	40	Carrageenan Blend Additive	0.2	40
	Locust Bean Gum	0.2	40	Locust Bean Gum	0.2	40
	Food Coloring (Red No.40)	0.1	20	Food Coloring (Red No.40)	0.1	20
	Natural disinfectants	0.05	10	Natural disinfectants	0.05	10
	Cocoa Color (JS-200)	0.025	5	Cocoa Color (JS-200)	0.025	5
	Purified Water	76.0750	15,215	Purified Water	68.575	13,715
	**Total net**	**100.00**	**20,000**	**Total net**	**100.00**	**20,000**

Table S3. The clinical safety test results in participants' serum in between test and control groups.

Variable	Observed value (FAS)			Change from baseline				
	Control (n=50)	Test (n=50)	*p*-value[**]	Control	*p*-value[*]	Test	*p*-value[*]	*p*-value[**]
**Hemoglobin**								
Start	13.05±1.67	13.11±1.19	0.842[1)]					
Terminal	13.47±1.06	13.26±1.13	0.344[1)]	0.41±1.11	**0.011**[2)]	0.15±0.55	**0.062**[2)]	0.132[1)]
**T-Cholesterol**								
Start	196.08±28.19	197.72±29.13	0.775[1)]					
Terminal	197.10±27.15	196.64±29.94	0.936[1)]	1.02±22.65	0.752[2)]	-1.08±12042	0.747[2)]	0.650[1)]
**Glucose**								
Start	89.68±7.83	88.76±8.62	0.578[1)]					
Terminal	89.62±7.94	89.40±9.34	0.899[1)]	-0.06±7.09	0.953[2)]	0.64±7.16	0.530[2)]	0.624[1)]
**Triglyceride**								
Start	101.72±60.66	85.20±35.10	0.100[1)]					
Terminal	99.04±54.86	93.30±75.96	0.666[1)]	-2.68±58.52	0.747[2)]	8.10±64.48	0.379[2)]	0.383[1)]
**SGOT**								
Start	23.44±6.31	23.42±6.07	0.987[1)]					
Terminal	22.00±6.24	22.02±5.63	0.987[1)]	-1.44±5.78	0.084[2)]	-1.40±5.41	0.073[2)]	0.972[1)]
**SGPT**								
Start	18.38±7.74	20.28±10.53	0.306[1)]					
Terminal	18.32±9.90	19.32±10.90	0.632[1)]	-0.06±8.06	0.958[2)]	-0.96±8.33	0.419[2)]	0.584[1)]
**γ-GTP**								
Start	31.92±26.85	22.48±10.09	**0.023**[1)]					
Terminal	31.96±26.08	24.88±14.04	0.094[1)]	0.04±9.44	0.976[2)]	2.40±8.20	**0.044**[2)]	0.328[1)]
**HDL-C**								
Start	60.98±12.37	61.68±11.20	0.767[1)]					
Terminal	62.18±14.43	62.08±11.71	0.970[1)]	1.20±5.95	0.160[2)]	0.40±7.85	0.720[2)]	0.567[1)]
**LDL-C**								
Start	113.72±29.20	116.82±29.55	0.599[1)]					
Terminal	114.40±27.89	115.12±26.35	0.895[1)]	0.68±20.22	0.813[2)]	-1.70±22.36	0.593[2)]	0.578[1)]
**Creatinine**								
Start	0.69±0.15	0.70±0.16	0.672[1)]					
Terminal	0.69±0.12	0.69±0.16	0.922[1)]	0.00±0.09	0.940[2)]	-0.01±0.09	0.387[2)]	0.577[1)]

Variable	Observed value (PP)			Change from baseline				
	Control (n=47)	Test (n=47)	*p*-value[**]	Control	*p*-value[*]	Test	*p*-value[*]	*p*-value[**]
**Diastolic BP** (mmHg)								
Start	70.89±12.63	67.68±13.27	0.232[1)]					
Terminal	71.11±13.43	67.15±11.67	0.131[1)]	0.21±9.93	0.884[2)]	-0.53±10.53	0.731[2)]	0.725[1)]
**Systolic BP** (mmHg)								
Start	120.45±16.15	114.81±15.99	0.092[1)]					
Terminal	119.17±15.41	114.74±14.91	0.160[1)]	-1.28±11.95	0.468[2)]	-0.06±13.41	0.974[2)]	0.645[1)]
**Pulse rate**								
Start	80.70±11.63	79.49±9.16	0.576[1)]					
Terminal	77.53±8.59	78.00±8.98	0.797[1)]	-3.17±9.16	**0.022**[2)]	-1.49±8.95	0.260[2)]	0.371[1)]

[*] *p-values* were compared within each group; [**] *p-values* were compared between groups; 1) Independent t-test; 2) paired t test.

Supplementary Figure legend

Supplementary Figure S1. Measurement of additional skin health indicators following GL administration in human clinical trial. (A-B) The comparison measurement of average wrinkle severity (Rmax and Rp) between the groups before and after sample intake. (C) The comparison measurement of average subsurface skin hydration rates between the groups before and after sample intake, as well as the changes in subsurface skin hydration rates from baseline for each group. (D) The comparison measurement of average subsurface TEWL rates between the groups before and after sample intake, as well as the changes in TEWL rates from baseline for each group. (E) The comparison measurement of average skin desquamation rates between the groups before and after sample intake, as well as the changes in skin desquamation rates from baseline for each group. (F) The comparison measurement of relative skin elasticity (R2) between the groups before and after sample intake. (G) The changes from baseline of relative skin elasticity (R5) in GL supplemented test and control groups. Data were presented to mean ± S.D. (n=47).

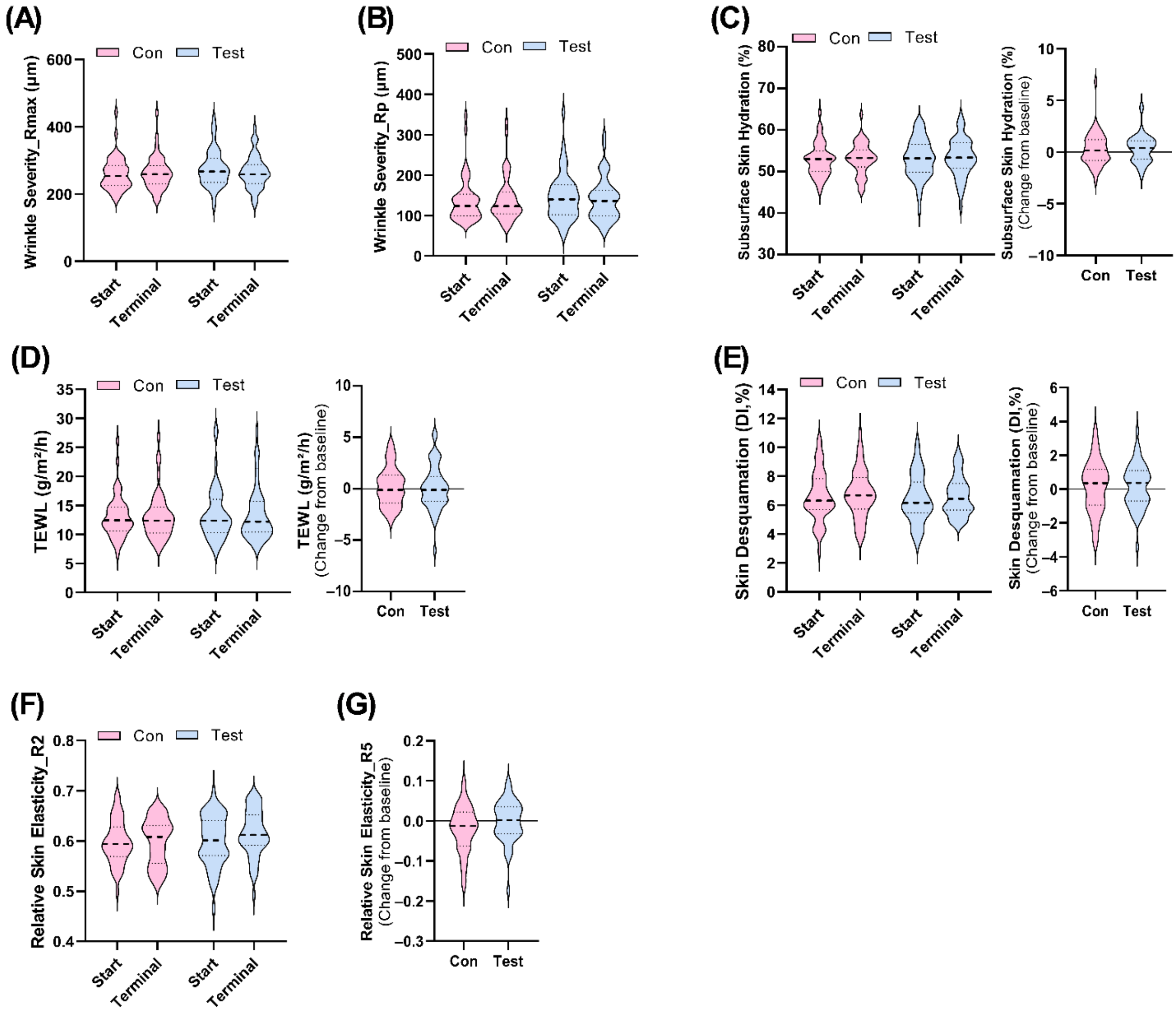

Hyun et al., Figure. S1.
